# Canine Leishmaniasis, Italy

**DOI:** 10.3201/eid1110.040966

**Published:** 2005-10

**Authors:** Ezio Ferroglio, Michele Maroli, Silvia Gastaldo, Walter Mignone, Luca Rossi

**Affiliations:** * University of Turin, Turin, Italy; † Istituto Superiore di Sanità, Rome, Italy; ‡ Istituto Zooprofilattico Sperimentale del Piemonte, Liguria e Valle d'Aosta, Imperia, Italy

**Keywords:** Leishmania, sand flies, dog, epidemiology, emerging diseases, new foci, seasonal dynamics, IFAT, north Italy, dispatch

## Abstract

We report the results of a survey to determine the prevalence of canine leishmaniasis and the presence of sand flies in northwestern Italy, where autochthonous foci of canine leishmaniasis have not been reported. Active foci of canine leishmaniasis were identified, which suggests that the disease is now also endemic in continental climate areas.

Visceral leishmaniasis due to *Leishmania infantum* is a zoonotic disease transmitted in Mediterranean areas by sand flies belonging to the genus *Phlebotomus*. Dogs are the main reservoir of infection, and in some disease-endemic areas seroprevalence of canine leishmaniasis is >30% (*1*). Recently, the geographic distribution of canine leishmaniasis has spread (*2*), and new foci of disease have been reported in countries such as the United States (*3*). Global warming is a possible cause of spread of the disease to cooler areas (*4*), and the increased movement of infected animals from areas where the disease has traditionally been endemic can, together with the spread of sand fly vectors, facilitate this process. In Europe, both canine leishmaniasis and human visceral leishmaniasis are endemic in Mediterranean areas characterized by a dry, hot summer and mild winter temperatures (*4*). However, foci of canine leishmaniasis have never been reported in continental climate regions, which are characterized by large seasonal temperature changes between hot summer and cold winters. Until recently, stable endemic foci of both human visceral leishmaniosis and canine leishmaniasis have been present only in southern, central, and insular regions in Italy. However, new foci of canine leishmaniasis and the presence of competent sand fly vectors have also been reported in northern regions of the country, where autochthonous cases had not been reported previously (*5**,**6*).

## The Study

A serologic survey for canine leishmaniasis was conducted in 3 areas of the Piedmont to study the spread of canine leishmaniasis in northwestern Italy and to establish if newly identified foci were stable. Study areas included hill zones close to Turin, Casale, and Ivrea, which are all characterized by a continental climate. Autochthonous cases of canine leishmaniasis had been increasingly reported by practitioners in these areas during the late 1990s, and a focus was recently identified in Turin (*6*). The survey was also extended to the neighboring Aosta Valley, a mountain region where autochthonous cases of canine leishmaniasis have not been reported or suspected. An entomologic survey was also conducted during 2 consecutive years (2000–2001) in the above areas to assess the presence of *Leishmania* vectors and to study their density and their seasonal dynamics. Sand flies were absent in both the Piedmont and Aosta Valley in a survey conducted 30 years previously (*7*).

Blood samples were collected from 913 asymptomatic resident dogs (that had never traveled to traditionally leishmaniasis-endemic areas) during the winter and spring months from January 1999 to March 2001. The tested dogs included 313 dogs from the surroundings of Turin (45.4°N, 7.70°E), 176 from Casale (45.8°N, 8.26°E) 155 from Ivrea (45.28°N, 7.52°E), and 269 from the Aosta Valley (45.4°N, 7.20°E). Serum samples were tested with the indirect fluorescent antibody test (IFAT), as previously described (*8*). Titers >1:160 were considered positive, values <1:40 were considered negative, and a value of 1:80 was considered doubtful (*9*). Prevalence values in the 5 study areas were compared by a chi-square test (EpiInfo, version 6.0, Centers for Disease Control and Prevention, Atlanta, GA, USA), and differences were considered significant when p was <0.05.

For the entomologic survey, 518 collecting sites containing a variety of sand fly diurnal resting sites (animal shelters, houses, and scarp wall cracks) were selected throughout the Piedmont and Aosta Valley regions: 146 in Ivrea, 89 around Casale, 194 near Turin, and 89 in Aosta. The presence or absence of *Leishmania* vectors was assessed once per collecting site from the second half of June to the first half of August, when major sand fly densities are expected (*6*). In addition, to evaluate sand fly seasonal dynamics, weekly collections were carried out from the end of April to the end of October 2001 in a few representative sites: 8 in Turin, 8 in Ivrea, and 6 in Aosta. Sand fly specimens were captured by sticky traps made from 20 × 20-cm, castor-oiled paper (*10*); a minimum of 10 sticky traps were used in each collecting site.

Sand flies were identified to the species level (*11*). Dogs with unambiguously positive serology (IFAT titers >1:160) were detected in all 4 examined areas. Seroprevalence did not differ significantly in Turin (4.5%), Ivrea (5.8%), and Casale (3.9%) but was significantly lower in Aosta (0.4%) (chi square = 25.6, p = 0.00004). In Turin, Ivrea, and Casale, infection was observed in resident autochthonous dogs in both 2000 and 2001, and in these areas the observed seroprevalences were higher than the 2.5% threshold usually associated with steadily established canine leishmaniasis foci (*12*). *Leishmania* amastigotes were also observed in lymph node smears from resident ill dogs from all 4 areas (E. Ferroglio, unpub. data).

Of 518 stations examined, 113 (21.8%) were positive for *Phlebotomus perniciosus* (Figure), the main vector of leishmaniasis in Italy (*13*), while *P. neglectus*, another recognized vector of canine leishmaniasis (*14*), was found only in Ivrea (Table). The percentage of positive stations did not significantly differ in the 4 areas.

**Figure:**
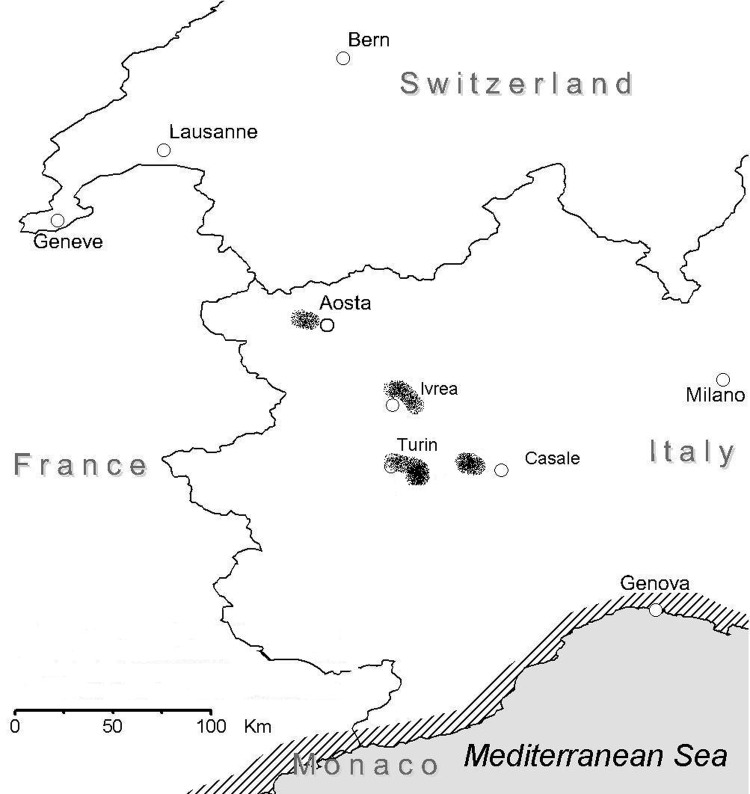
Traditionally endemic canine leishmaniosis (canine leishmaniasis) areas (slash marks) and new foci in continental climate areas of northwestern Italy (shaded areas).

**Table:** Sand flies collected in 4 study areas, 2000–2001

Study area	*Phlebotomus perniciosus*, no. (% positive stations)	*P. neglectus*, no. (% positive stations)	*P. mascittii*, no. (% positiv stationse)	*Sergentomya minuta*, no. (% positive stations)
Ivrea	28 (12)	37 (3.3)	2 (1.4)	1,162 (43.3)
Casale	39 (40.4)	0	1 (1.1)	1,708 (34.7)
Aosta	136 (28)	0	3 (3.4)	1,265 (41)
Turin	225 (20.8)	0	1 (0.4)	857 (79.2)
Total	428 (23.2)	37 (1.0)	7 (1.8)	4,992 (36.0)

In 2001, *P. perniciosus* was first captured in Turin on May 11 and during the last week of May in the remaining 2 areas. The last captures occurred in Aosta during the last 10 days of September and in Turin and Ivrea during the first 10 days of October. Peak numbers of *P. perniciosus* were observed in Turin (10.6 individuals/m^2^ sticky traps) and Ivrea (7.3/m^2^) during the last 10 days of July. Peak density in Aosta was lower (2.5/m^2^) and slightly earlier (first 10 days of July).

## Conclusions

Our data indicate that canine leishmaniasis is now endemic in at least 3 different areas of the Piedmont (Turin, Ivrea, and Casale), where seroprevalence in resident dogs is 3.9%–5.8%. A possible unstable focus has been identified in the Aosta Valley. Of 518 stations examined, 113 (21.8%) were positive for *P. perniciosus* (Table). The percentage of positive stations did not significantly differ in the 4 areas. In the Aosta Valley, 25 (28.0%) of 89 stations examined were positive for *P. perniciosus*. In this mountain area, sand flies have not been reported previously, and 23 sticky trap capture stations monitored in the late 1960s were all negative (*7*). Colonization of these areas may have occurred either spontaneously, from Mediterranean coastal areas, or following the increased movement of people towards Mediterranean areas, where phlebotomine sand flies are abundant. The seasonal presence of sand flies extends from the second half of May to September. These results may be usefully exploited to define the risk period for canine (and human) leishmaniasis transmission in northern Italy. Recently, a preliminary survey conducted in northwestern Italy showed *L. infantum* infection in the resident human population (*15*), and polymerase chain reaction–restriction fragment length polymorphism analysis of the human and canine strains provided evidence of the circulation of *L. infantum* between dogs and humans in this area (E. Ferroglio et al., unpub data). Our findings are evidence that canine leishmaniasis is currently expanding in continental climate areas of northwestern Italy, far from the recognized disease-endemic areas along the Mediterranean coasts (Figure). Based on similarities in climate and major landscape features with our study area, spread of canine leishmaniasis to other regions of central Europe can be foreseen in the near future.
